# Evidence of a Shift in the Littoral Fish Community of the Sacramento-San Joaquin Delta

**DOI:** 10.1371/journal.pone.0170683

**Published:** 2017-01-24

**Authors:** Brian Mahardja, Mary Jade Farruggia, Brian Schreier, Ted Sommer

**Affiliations:** California Department of Water Resources, Division of Environmental Services, West Sacramento, California, United States of America; Havforskningsinstituttet, NORWAY

## Abstract

Many estuarine and freshwater ecosystems worldwide have undergone substantial changes due to multiple anthropogenic stressors. Over the past two decades, the Sacramento-San Joaquin Delta (Delta) in California, USA, saw a severe decline in pelagic fishes, a shift in zooplankton community composition, and a rapid expansion of invasive aquatic vegetation. To evaluate whether major changes have also occurred in the littoral fish community, we analyzed a beach seine survey dataset collected from 1995 to 2015 from 26 sites within the Delta. We examined changes in the Delta fish community at three different ecological scales (species, community, and biomass), using clustering analyses, trend tests, and change-point analyses. We found that the annual catch per effort for many introduced species and some native species have increased since 1995, while few experienced a decline. We also observed a steady pattern of change over time in annual fish community composition, driven primarily by a steady increase in non-native Centrarchid species. Lastly, we found that littoral fish biomass has essentially doubled over the 21-year study period, with Mississippi Silverside *Menidia audens* and fishes in the Centrarchidae family driving most of this increase. The changes in the catch per effort, fish community composition, and biomass per volume indicate that a shift has occurred in the Delta littoral fish community and that the same factors affecting the Delta’s pelagic food web may have been a key driver of change.

## Introduction

Ecosystem shifts are often large-scale, abrupt, and can cause persistent ecological changes [1,2]. Most recorded ecosystem shifts have been driven by direct anthropogenic pressures [3,4], and have led to substantial changes in the provision of ecosystem services with significant effects on human well-being and resources [5]. However, ecosystem shifts can be difficult to detect as they can unfold slowly until a tipping point has been exceeded [6]. Upon reaching a new stable state, these changes may be very difficult or almost impossible to reverse [2,7]. Even when such ecosystem shifts are reversible, the cost and time required to reverse the changes can be prohibitive without prompt and early intervention [8,9,10]. Thus, it is critical for resource managers to document and fully understand ecosystem shifts as they occur.

**Fig 1:**
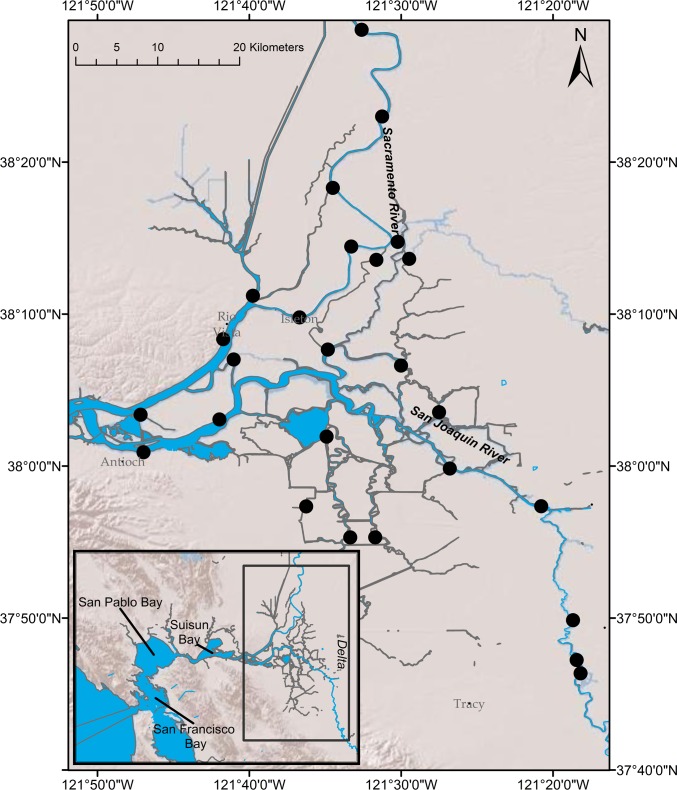
Study area map denoting locations of beach seine survey stations used in the study.

The San Francisco Estuary, a large estuary located on the Pacific coast of United States in California, has experienced a series of well-described changes to its physical conditions and biota. Since the mid-nineteenth century, the San Francisco Estuary has undergone major alterations that include extensive wetland removal, flow diversions, and introductions of various invasive species [11,12]. As such, the San Francisco Estuary has earned a reputation as one of the most highly managed and invaded estuaries in the world [13,14]. In recent years, much attention has been paid to the upper San Francisco Estuary (brackish to freshwater portion of the estuary) ecosystem due to a putative shift that has significant social, economic, and ecological consequences [15]. Although the pelagic fish community of the upper San Francisco Estuary has historically shown substantial variability in abundance, several pelagic fish species experienced a severe collapse in the early 2000s [12,16]. This decline in pelagic fish species abundance is referred to regionally as the Pelagic Organism Decline (POD) [12,17] and the underlying mechanisms for this shift has been a key area of research [18,19,20]. Over a similar timeframe as the POD, there was a notable shift in zooplankton species composition throughout the upper San Francisco Estuary with little change to the overall zooplankton biomass [21]. These changes were accompanied by further expansion of introduced submerged aquatic macrophytes [22,23] and fish species [24,25] in the Sacramento-San Joaquin Delta (Delta), which is the upstream, tidal freshwater portion of the San Francisco Estuary (Fig 1).

Most, if not all, ecosystem shifts constitute a food web reorganization in which the decline of certain species coincide with the increase of other species [26,27,28]. Previous studies describing the POD shift in the upper San Francisco Estuary have generally focused on the declining pelagic fish species [16,18,20,29,30,31] that occupy open water habitat and exhibit relatively unique life histories [32]. The few analyses on Delta littoral fish species indicated that major shifts have also occurred in the littoral habitat [24,25,33]. However, such studies are scarce and lack a continuous time series dataset [24] or are more focused on the spatial variation in fish species composition [34,35]. Moreover, none of the studies to date have attempted to evaluate changes at multiple ecological scales (*e*.*g*. species, community, biomass). A comprehensive description of responses in littoral fish community (if any) is needed in order to fully identify and characterize all the changes associated with the POD.

The San Francisco Estuary is a relatively well-sampled system, with multiple long-term aquatic monitoring programs spanning many decades. Among these monitoring programs is the Delta Juvenile Fish Monitoring Program (DJFMP) beach seine survey conducted by the United States Fish and Wildlife Service, which samples smaller littoral fishes around the San Francisco Estuary and its watershed, including the Delta. The DJFMP beach seine survey has been ongoing since the 1970s and has been expanded several times to cover a broader geographic range and improve temporal resolution.

Long-term time series datasets are essential for the evaluation of temporal trends and for describing changes associated with an ecosystem shift [36]. Long-term datasets that capture a wide range of species are especially useful as they allow researchers to better characterize the community dynamics over time, and thus, can be crucial in understanding the effects of environmental change and management actions. The objective of this study was to describe whether the Delta littoral fish community changed during the prominent ecosystem shift that is the POD. To reach this objective, we examined the DJFMP beach seine survey dataset at three ecological scales: species, community, and biomass. We reasoned that examining three ecological scales would provide a more comprehensive representation of the ecological changes for the littoral fish community. Specifically, we evaluated the following: (1) whether any species exhibit strong increasing or decreasing trends over the study period, (2) if composition of fish community has changed, and (3) if there has been a shift in fish biomass over time. Our hope was that these results would provide insight into how the long-term ecological shifts in the San Francisco Estuary have affected the Delta littoral fish community.

## Materials and Methods

### Study Area

The San Francisco Estuary is one of the largest and most well-studied estuaries along the Pacific coast of the United States [17]. The system is located in central California and is characterized by a Mediterranean climate of high precipitation in winter-spring and dry weather in summer-fall (Fig 1). At the most upstream region of the San Francisco Estuary is the Delta, a tidal freshwater channel network shaped by the confluence of the two largest rivers in California—the Sacramento (from the north) and the San Joaquin (from the south). Though once a dynamic system of tidal floodplains and marshes formed by the Sacramento and San Joaquin Rivers, the Delta’s wetlands have been diked and filled extensively over the past two hundred years. Today, the Delta exists as a network of highly modified waterways with over 1,000 miles of levees [37,38]. The Delta today serves as a major hub of California’s water supply, conveying water from the wetter Northern California region watersheds to millions of California’s households and millions of acres of farmlands to the south [37].

### Data Source

Sampling for the DJFMP beach seine survey began in 1976 with the original goal of monitoring the abundance and distribution of juvenile Chinook Salmon (*Oncorhynchus tshawytscha*) in the Delta and upstream [39]. Since then, the survey has been expanded several times but has remained focused on the Delta region and nearby locations. Over the years, it has collected substantial information on a number of littoral fish species [25,35,40,41]. Although the DJFMP was initiated in the 1970s, consistent sampling in spring and summer months within the Delta region did not begin until 1995. Since 1995, the DJFMP has sampled 26 sites within the Delta either weekly or biweekly (twice a month) year-round (Fig 1) with the occasional exceptions of when sampling is prohibitive due to various logistical reasons (*e*.*g*. lack of a beach due to high tides or low flows, obstructions within sampling site, lack of personnel, unsafe conditions) (S1 Fig, S1 Table). Sampling consisted of hauling a 15.2 m x 1.3 m beach seine net with 3 mm mesh and a 1.3 m x 1.3 m bag. Nets had float and lead lines attached to 1.8 m poles at both ends. Volume of water sampled by the beach seine was estimated for each sampling event via measurements of the length, width, and mean depth of the location sampled. After each seine haul, all fish were identified to species (for fish greater than 25 mm) and then counted. Up to 30 fish per species from each sample were measured for fork lengths (FL, in mm) after which any additional fish were simply counted. For fish species that have been listed under the Endangered Species Act, up to 50 fish per species were measured.

We limited our analysis to the aforementioned 26 Delta sites to maximize the consistency of sampling effort throughout the study period (1995 to 2015). Additionally, we only used data collected between the months of March and August because most Delta fish species spawn in the spring or summer [32,35] and thus would be present at highest numbers. Salmonid species (Chinook Salmon and Steelhead Trout) were excluded from our analyses due to the confounding effects of fish stocking upstream of the Delta [42,43].

### Data Analysis

#### 1. Species abundance trends

We limited our analysis to 23 species that had both over roughly 1,000 individuals caught during the study period and no more than one year with zero catch because abundance patterns are unlikely to be accurate for rare fishes (S2 Table). Fish count data were first adjusted by volume in cubic meter (hereafter “catch per effort”) and averaged by month, site, and species. Each species’ mean monthly catch per effort was then averaged across the 26 Delta sites. Annual catch per effort of each species was then finally calculated as the mean of these Delta-wide average monthly catch per effort. To see if each fish species had experienced a long-term shift in catch per effort numbers or remain the same, we applied the Mann-Kendall test for monotonic temporal trend [44] on the annual mean catch per effort time series dataset for the 23 species. To further confirm the Mann-Kendall test results, we also applied the Pettitt’s test for single change-point detection on the same dataset [45]. The program R [46] was used to conduct both tests: “Kendall” package for the Mann-Kendall test [47] and “trend” package for Pettitt’s test [48]. The significance level for both tests was evaluated at α = 0.05 using Benjamini and Hochberg’s [49] method for controlling false discovery rate in multiple testing.

#### 2. Community dynamics

We chose species with at least 100 individuals caught over the study period for the community level analysis, resulting in a total of 31 fish species (S2 Table). Species mean annual catch per effort was fourth-root transformed to reduce overrepresentation of species with exceedingly high catch numbers (*i*.*e*. Mississippi Silverside *Menidia audens*) (S2 Table) [50]. Ordination of annual catch data was conducted by using the “vegan” package in R [51]. We first calculated the Bray-Curtis dissimilarity index for each year comparison and then plotted the results in two dimensions using the non-metric multidimensional scaling (NMDS) method [52]. NMDS stress value was evaluated to ensure that the input data is well represented in the final two-dimensional figure according to Clarke’s [50] guidelines. Samples (years) in the resulting NMDS plot were color coded based on the California Department of Water Resources’ Sacramento Valley water year index (http://cdec.water.ca.gov/cgi-progs/iodir/WSIHIST) to help visualize how freshwater inflow affected littoral fish community composition in the Delta. Correlation vectors to species fourth-root transformed catch per effort numbers were calculated using “envfit” function in the “vegan” R package [51]. The resulting correlation vectors with high R^2^ (>0.4) and low *p*-values (<0.01) were plotted onto the NMDS figure. Four species vectors (Smallmouth Bass *Micropterus dolomieu*, Spotted Bass *Micropterus punctulatus*, Threespine Stickleback *Gasterosteus aculeatus*, Western Mosquitofish *Gambusia affinis*) were excluded in the final figure to reduce the complexity of the plot and ease its interpretation (for NMDS figure with all vectors plotted, see S2 Fig).

To identify the temporal shift or change in fish community, we conducted hierarchical clustering analysis in conjunction with NMDS by way of similarity profile (SIMPROF) testing [53]. The SIMPROF test allows for the evaluation of structuring among samples without requiring *a priori* factors. If a temporal shift in the littoral fish community did not occur, we would expect samples (*i*.*e*. years) to cluster by environmental drivers (*e*.*g*. freshwater input) rather than by proximity in time. We performed the SIMPROF test by using the “clustsig” package in R [54] with 1,000 permutations, group average agglomeration method, and an α of 0.05. To determine which species contribute most to fish community differences between clusters of years, we conducted similarity percentage (SIMPER) analyses [50] on the groups identified by SIMPROF.

To further confirm the presence of a temporal shift in community composition and to aid the interpretation of the NMDS plot, Mann-Kendall tests were performed on the two NMDS axis scores with an α of 0.05. Kendall rank correlation tests were also conducted between the average March to August freshwater inflow into the Delta and the two NMDS axis scores (α = 0.05). Average inflow was calculated in cubic feet per second and was acquired from the DAYFLOW dataset (http://www.water.ca.gov/dayflow/).

#### 3. Biomass

We estimated the biomass for each species by applying length-weight equations found in the literature to the fork length data collected by the DJFMP beach seine survey (Table 1). When possible, we used species length-weight equations acquired from data collected in the San Francisco Estuary [55]. For those species not included in Kimmerer et al. [55], we applied the species length-weight equations found in Schneider et al. [56], which used total length measurements in place of fork length. The sole exception was for the Sacramento Pikeminnow (*Ptychocheilus grandis*), in which we used the length-weight equation from Nobriga et al. [57]. Because total length measurements were used in Schneider et al. [56], there will be underestimation of biomass for a portion of the species we analyzed. Each species was classified by their status (native vs. introduced) and the habitat type that it is most associated with (*e*.*g*. pelagic, benthic, or littoral) based on information in Moyle [32].

**Table 1:** List of species used for biomass per volume analysis with their respective equations, habitat type association, and native/introduced status based on information in Moyle [32].

Common Name	Species	Length-Weight Equation Used	Habitat	Native?
American Shad	*Alosa supidissima*	Kimmerer et al. 2005 (American Shad)	Pelagic	No
Bass, unknown	*Micropterus spp*.	Schneider et al. 2000 (Largemouth Bass)	Littoral	No
Bigscale Logperch	*Percina macrolepida*	Schneider et al. 2000 (Blackside Darter)	Littoral	No
Black Bullhead	*Ameiurus melas*	Schneider et al. 2000 (Bullhead)	Benthic	No
Black Crappie	*Pomoxis nigromaculatus*	Schneider et al. 2000 (Black Crappie)	Littoral	No
Bluegill	*Lepomis macrochirus*	Schneider et al. 2000 (Bluegill)	Littoral	No
Brown Bullhead	*Ameiurus nebulosus*	Schneider et al. 2000 (Bullhead)	Benthic	No
California Roach	*Hesperoleucus symmetricus*	Kimmerer et al. 2005 (Splittail)	Littoral	Yes
Chameleon Goby	*Tridentiger trigonocephalus*	Kimmerer et al. 2005 (Shimofuri Goby)	Benthic	No
Channel Catfish	*Ictalurus punctatus*	Schneider et al. 2000 (Channel Catfish)	Benthic	No
Common Carp	*Cyprinus carpio*	Kimmerer et al. 2005 (Common Carp)	Littoral	No
Delta Smelt	*Hypomesus transpacificus*	Kimmerer et al. 2005 (Delta Smelt)	Pelagic	Yes
Fathead Minnow	*Pimephales promelas*	Schneider et al. 2000 (Golden Shiner)	Littoral	No
Golden Shiner	*Notemigonus crysoleucas*	Schneider et al. 2000 (Golden Shiner)	Littoral	No
Goldfish	*Carassius auratus*	Kimmerer et al. 2005 (Common Carp)	Littoral	No
Green Sunfish	*Lepomis cyanellus*	Schneider et al. 2000 (Green Sunfish)	Littoral	No
Hardhead	*Mylopharodon conocephalus*	Kimmerer et al. 2005 (Splittail)	Littoral	Yes
Hitch	*Lavinia exilicauda*	Kimmerer et al. 2005 (Splittail)	Littoral	Yes
Mississippi Silverside	*Menidia audens*	Kimmerer et al. 2005 (Silverside)	Littoral	No
Largemouth Bass	*Micropterus salmoides*	Schneider et al. 2000 (Largemouth Bass)	Littoral	No
Longfin Smelt	*Spirinchus thaleichthys*	Kimmerer et al. 2005 (Longfin Smelt)	Pelagic	Yes
Pacific Herring	*Clupea pallasii*	Kimmerer et al. 2005 (Pacific Herring)	Pelagic	Yes
Pacific Staghorn Sculpin	*Leptocottus armatus*	Kimmerer et al. 2005 (Pacific Staghorn Sculpin)	Benthic	Yes
Prickly Sculpin	*Cottus asper*	Kimmerer et al. 2005 (Prickly Sculpin)	Benthic	Yes
Rainwater Killifish	*Lucania parva*	Kimmerer et al. 2005 (Rainwater Killifish)	Littoral	No
Red Shiner	*Cyprinella lutrensis*	Schneider et al. 2000 (Golden Shiner)	Littoral	No
Redear Sunfish	*Lepomis microlophus*	Schneider et al. 2000 (Redear Sunfish)	Littoral	No
Redeye Bass	*Micropterus coosae*	Schneider et al. 2000 (Largemouth Bass)	Littoral	No
Sacramento Blackfish	*Orthodon microlepidotus*	Kimmerer et al. 2005 (Splittail)	Littoral	Yes
Sacramento Pikeminnow	*Ptychocheilus grandis*	Parker et al. 1995 (Combined male and female regression from downstream of Bonneville Dam)	Littoral	Yes
Sacramento Sucker	*Catostomus occidentalis*	Kimmerer et al. 2005 (Sacramento Sucker)	Benthic	Yes
Shimofuri Goby	*Tridentiger bifasciatus*	Kimmerer et al. 2005 (Shimofuri Goby)	Benthic	No
Shokihaze Goby	*Tridentiger barbatus*	Kimmerer et al. 2005 (Shimofuri Goby)	Benthic	No
Smallmouth Bass	*Micropterus dolomieu*	Schneider et al. 2000 (Smallmouth Bass)	Littoral	No
Splittail	*Pogonichthys macrolepidotus*	Kimmerer et al. 2005 (Splittail)	Littoral	Yes
Spotted Bass	*Micropterus punctulatus*	Schneider et al. 2000 (Smallmouth Bass)	Littoral	No
Starry Flounder	*Platichthys stellatus*	Kimmerer et al. 2005 (Starry Flounder)	Benthic	Yes
Striped Bass	*Morone saxatilis*	Kimmerer et al. 2005 (Striped Bass)	Pelagic	No
Threadfin Shad	*Dorosoma petenense*	Kimmerer et al. 2005 (Threadfin Shad)	Pelagic	No
Three Spine Stickleback	*Gasterosteus aculeatus*	Kimmerer et al. 2005 (Three Spine Stickleback)	Littoral	Yes
Tule Perch	*Hysterocarpus traskii*	Kimmerer et al. 2005 (Tule Perch)	Littoral	Yes
Wakasagi	*Hypomesus nipponensis*	Kimmerer et al. 2005 (Delta Smelt)	Pelagic	No
Warmouth	*Lepomis gulosus*	Schneider et al. 2000 (Warmouth)	Littoral	No
Western Mosquitofish	*Gambusia affinis*	Kimmerer et al. 2005 (Western Mosquitofish)	Littoral	No
White Catfish	*Ameiurus catus*	Schneider et al. 2000 (Channel Catfish)	Benthic	No
White Crappie	*Pomoxis annularis*	Schneider et al. 2000 (White Crappie)	Littoral	No
Yellowfin Goby	*Acanthogobius flavimanus*	Kimmerer et al. 2005 (Yellowfin Goby)	Benthic	No

Because not all fish are measured for length, species biomass for each sample (*M*) was calculated as follows:

$$M=\frac{\left(n+c\right)}{n}\sum_{i=1}^{n}\beta_{0}{{(l}_{i})}^{\beta_{1}}$$

Where *n* is the number of fish measured in a sample, *c* is the number of unmeasured fish in the sample, *l*_*i*_ is the length of fish *i*, and *β*_0_ and *β*_1_ are the length-weight regression coefficients used for the species. Each species biomass or weight per sample was then divided by the sample volume (in m^3^) to acquire species biomass per volume. Species annual mean biomass per volume was calculated by averaging monthly means across all sites in the same manner as the species annual catch per effort calculation. To identify if a shift had occurred at the total biomass level, we used the Mann-Kendall and Pettitt’s tests similar to our species abundance trend analysis.

## Results

### 1. Species Abundance Trends

**Fig 2:**
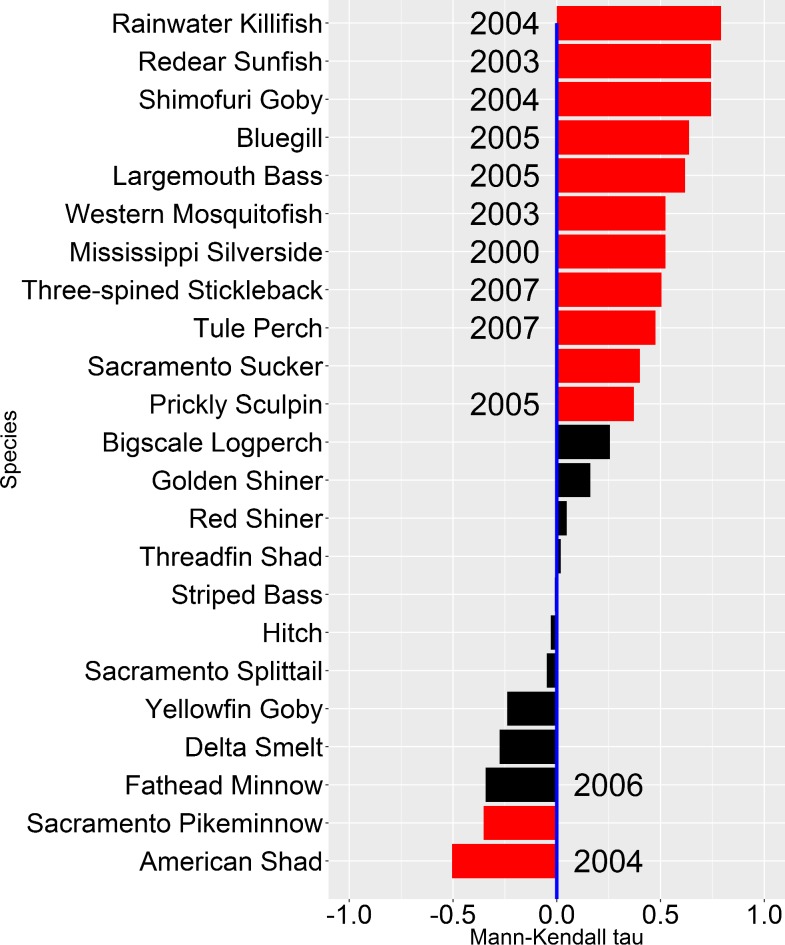
Catch per effort trends for the study period (1995–2015) of 23 fish species in the Sacramento-San Joaquin Delta. Red bars indicate significance for Mann-Kendall test at *α* = 0.05. Year next to bar indicates the year assigned as change point based on Pettitt’s test at *α* = 0.05. Lack of year next to bar indicates non-significant Pettitt’s test for the species.

We detected a significant monotonic trend in species annual catch per effort between 1995 and 2015 for 13 of 23 fish species analyzed (Mann-Kendall test; Fig 2, S3 Table). Of the species analyzed, eleven species were found to have increased over the 21-year period, two species had declined, and no apparent trends were detected for the remaining ten species. A change point was detected for 12 out of 23 species based on Pettitt’s test (Fig 2, S4 Table), though note that type I and II error rates for the test may be high when the dataset is highly variable or when the true change point occurs close to either ends of the time series [45]. Of the 13 species that tested significant for the presence of a monotonic trend, eleven also tested significant for the presence of a change point (Fig 2). The seven species with the highest Mann-Kendall tau coefficients are all introduced species, while the next four (Threespine Stickleback, Tule Perch, Sacramento Sucker, and Prickly Sculpin) are native species. Of the two species with significant negative Mann-Kendall tau coefficients (*i*.*e*. declining abundance trend), one species is non-native (American Shad), and the other (Sacramento Pikeminnow) is native.

### 2. Community Dynamics

**Fig 3:**
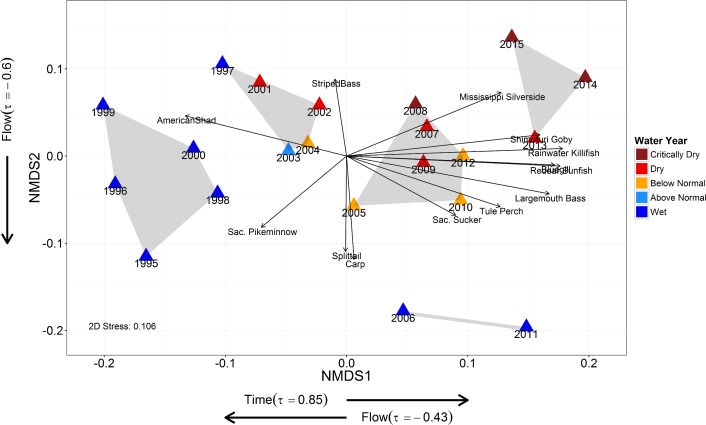
NMDS plot of fourth-root transformed catch per effort for fish species in the Sacramento-San Joaquin Delta. Arrows indicate general direction of increasing species abundance. Only species possessing correlation vectors with high R^2^ (>0.4) and low p-value (<0.01) are shown. Grey shading indicates significant SIMPROF cluster based on 1,000 permutations. Significant results for Mann-Kendall trend test and Kendall rank correlation tests are shown (α = 0.05).

The two-dimensional NMDS plot adequately represented the fourth-root transformed catch data input based on the low stress value at 0.106 (Fig 3). The Mann-Kendall test for temporal trend was significant for NMDS axis 1 (*p* < 0.001), but not for NMDS axis 2 (*p* = 0.61). Kendall rank correlation test with Delta freshwater inflow were significant for both NMDS axis 1 and 2 (*p* < 0.001 and *p* < 0.01, respectively). NMDS axis 1 generally depicted a temporal progression from the late 1990s to 2015 (*τ* = 0.85); though this trend was also accompanied by a partial progression towards lower freshwater inflow between 1995 and 2015 (*τ* = -0.43). NMDS axis 2 appeared to largely capture fish community differences based on freshwater input, with lower (i.e. negative) NMDS values being more associated with high flow years and higher NMDS values being more associated with low flow years (*τ* = -0.60) (S2 Fig). The SIMPROF test identified five statistically significant clusters of years, with the most recent wet years of 2006 and 2011 being the most differentiated group (S3 Fig).

Based on our SIMPER analysis, the two SIMPROF clusters containing only wet years (cluster A and D in S5 Table under Supporting Information) were generally characterized by increased catches of Sacramento Splittail and a reduction in Mississippi Silverside numbers (S5 and S6 Tables). Conversely, SIMPROF clusters that included dry years were characterized mainly by a higher numbers of Mississippi Silversides and lower numbers of Sacramento Splittail. The cluster which contained the two most recent wet years (2006 and 2011) was distinguished from the wet years of late 1990s by the comparatively higher catch of Sacramento Splittail, Common Carp, Rainwater Killifish, and Largemouth Bass (A-D comparison in S6 Table). Meanwhile, the most recent drought years (2013–2015) were differentiated from previous dry years by their relatively lower numbers of Sacramento Splittail and Red Shiner, as well as their increase in Rainwater Killifish catches (C-E comparison in S6 Table).

### 3. Biomass

**Fig 4:**
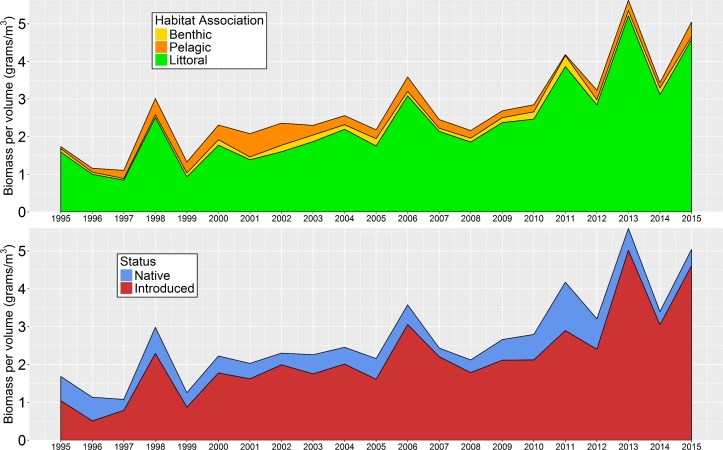
Estimated annual mean biomass per volume of all fish grouped by habitat association (top) and native-introduced status (bottom) between 1995 and 2015. This figure demonstrated that the biomass increase pattern was not a byproduct of the pelagic or benthic habitat, and that it was driven mainly by alien or introduced species.

**Fig 5:**
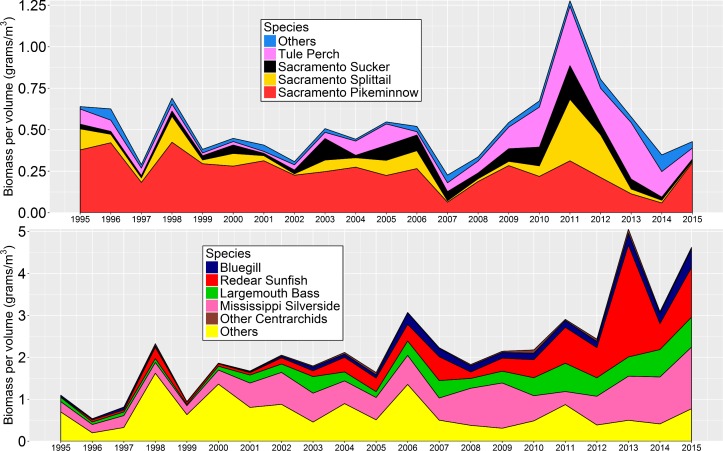
Estimated annual mean biomass per volume for native fishes (top) vs. non-native (i.e. introduced) fishes (bottom).

There was a significant increasing trend in total fish biomass per volume within the DJFMP beach seine survey dataset over the study period (Fig 4). The Mann-Kendall test for overall fish biomass per volume was significant at *p* <0.001 with a tau coefficient of 0.629. Pettitt’s test was also significant at *p* <0.05, denoting a change point in 2005. Littoral fish species collectively made up 85.5% of the total biomass per volume within the study period, suggesting that the increases in biomass per volume were not a byproduct of the pelagic or benthic fish community which happened to be captured by the beach seine survey. The majority of the biomass observed was comprised of introduced species, as they made up 79.8% of the total biomass per volume within the study period. Of the introduced species, Mississippi Silversides and species within the Centrarchidae family made up a large portion of the total biomass per volume over the study period at 33.3% and 22.9% respectively (Fig 5).

## Discussion

The observed responses in species catch per effort, community composition, and biomass per volume in the DJFMP beach seine survey indicate that the littoral fish community of the Delta has changed substantially since the late 1990s. The Delta littoral fish community is currently dominated by large numbers of Mississippi Silverside and Centrarchid species, with a doubling of their biomass since 1995. These changes likely reflected the same ecosystem shift as the POD, given that the changes were observed occurred within a similar timeframe as the POD and several other associated shifts to the biota of the region [12,16,21]. As such, our results suggest that the environmental drivers that caused the POD may have also been largely beneficial to the littoral fish community; in particular, introduced fish species. However, we acknowledge that our results do not resemble an abrupt ecosystem shift that is more typically examined in the ecological literature [1,36,58]. Moreover, it is uncertain whether the ecosystem shift we observed is relevant for all littoral habitats in the region.

Although the specific mechanism for the increase in Delta littoral fish catch per effort and biomass per volume is unclear, the remarkable invasion of aquatic macrophytes in littoral habitats of the Delta have likely enhanced primary productivity in these habitats [22,23]. Recent studies in the upper San Francisco Estuary region showed that when aquatic macrophytes are present, they can be an important contributor to the food web for littoral fishes [59,60]. In contrast, the pelagic fishes associated with the POD tend to consume organisms derived largely from phytoplankton-based production [60,61]. Since 1987, phytoplankton biomass in the upper San Francisco Estuary declined substantially due to the invasion of the “overbite” clam *Potamocorbula amurensis* [62,63,64]. Further spread of submerged and floating aquatic macrophytes in recent years may have exacerbated the pelagic fish food deficiency by limiting light availability for phytoplankton in the Delta [65]. However, we note that Delta pelagic and littoral fish species possess fairly diverse life histories and as such, it is likely that the expansion of aquatic macrophytes is just one of several factors responsible for changes in fish biomass in pelagic and littoral habitats.

### 1. Species Abundance Trends

Most of the fish species encountered in this study showed increase in their beach seine catch per effort numbers over the study period (Fig 2 and S4 Fig). Introduced fishes showed the most consistent increases over time, though the key factors behind the increases likely differ between species. For example, previous studies in the Delta [33] and elsewhere [66,67,68,69] have shown a positive relationship between submerged aquatic macrophytes and fish species within the Centrarchidae family (*e*.*g*. Largemouth Bass, Bluegill, Redear Sunfish). This association indicates that the recent proliferation of invasive macrophytes in the Delta [22,23] directly facilitated the increase of Centrarchid species. By contrast, increase in Mississippi Silversides may be driven more by the high frequency of drought years since the late 2000s based on their close association with low flow periods [25].

Although many native fish species within the Delta are presumed to be highly vulnerable to extinction due to habitat alterations and climate change [70], we found evidence that a few native fishes such as the Sacramento Sucker and Tule Perch have increased in numbers within littoral habitats since the 1990s. Brown and Michniuk [24] found that Tule Perch catch in electrofishing surveys declined from 1980s to the early 2000s, but a more recent electrofishing survey in 2010–2011 found Tule Perch in even higher numbers than that found in the 1980s for a localized region around the western Delta [60]. The agreement between our finding and the recent electrofishing survey [60] suggests that there was a true abundance increase of Tule Perch in the Delta in recent years. Nonetheless, the patterns of increases for Tule Perch and Sacramento Sucker were not as consistent as the increases we observed for the introduced Centrarchids (Fig 2, S4 Fig, S3 Table).

Despite the general increase in overall biomass per volume and catch per effort for many fish species, American Shad and Sacramento Pikeminnow both showed evidence of declines (Fig 2 and S4 Fig). American Shad is generally considered a pelagic species, so its decline is consistent with changes associated with the POD. Meanwhile, lower freshwater flow into the Delta in recent years may play a role in the reduction of Sacramento Pikeminnow catch, since high flows appear to facilitate increased dispersal of their offspring into the Delta [57]. It is unclear whether the decline in Sacramento Pikeminnow catch reflects overall abundance trends for this species, but this pattern is consistent with the predictions that the species will utilize cooler upstream areas more often in the future [71].

### 2. Community Dynamics

The Delta littoral fish community varied considerably from year to year and has changed significantly from 1995 to 2015. Some of these annual differences in fish community composition appear to be driven by the interannual variability of freshwater inflow into the Delta, a pattern reflected in the second NMDS axis (Fig 3). This result is not surprising given that numerous studies have demonstrated a strong relationship between fish community and hydrologic variability in California [72,73,74,75]. Yet, the largest differentiation between years, as captured by the first NMDS axis, seems to follow a pattern of continuous change over time. We acknowledge that there was a negative correlation between time and freshwater inflow to the Delta over the study period (dry years were more common and extreme in the decade between 2005 and 2015). However, this correlation is imperfect, as the decade of 2005–2015 included two notably wet years (2006 and 2011) and each dry year was not always followed by an even drier year (Fig 3). Indeed, when our NMDS plot is compared to the plot of Delta freshwater inflow by year (S2 Fig), the two plots demonstrate similar overall patterns. We also reason that the clear differentiation between the more recent wet years (2006, 2011) and the wet years of late-1990s is further evidence that an ecosystem shift has occurred over the study period (see S5 and S6 Tables for further details on the fish community differences between these wet years).

### 3. Biomass

Despite some interannual variability in Delta littoral fish biomass, there has been a general increase in biomass per volume over the study period. Total biomass per volume in the Delta has doubled from just under 2 g/m^3^ in the late 1990s to over 4 g/m^3^ in 2013–2015, largely driven by increases in introduced species. The increase in biomass per volume is consistent with the high proportion of species increasing in abundance over the years (Fig 2). Nonetheless, the majority of this biomass increase was driven by just a handful of species from the Mississippi drainage of the United States [32] (Fig 5). Within our study period (1995–2015), over 50% of the total biomass per volume was comprised of Centrarchid species and Mississippi Silversides. For the Centrarchids, a majority of the biomass per volume came from only three species: Largemouth Bass, Bluegill, and Redear sunfish (Fig 5).

From a management perspective, our results seem especially troubling. As previously noted, a decline of fish and zooplankton in the pelagic habitat of the upper San Francisco Estuary occurred within a similar timeframe [12,16,21]. The concurrent nature of these changes therefore can be interpreted as a major shift in the ecosystem from a largely pelagic food web to a littoral one. The exact environmental drivers behind these changes are difficult to identify, but anthropogenic effects in the upper San Francisco estuary have been considerable and encompassed habitat modifications, water diversions, introductions of non-native species, and pollution [19]. Particularly, some of the substantial changes that occurred around the time of the POD included abrupt changes in sediment supply [23,76], alterations in pesticide use [77], nutrient inputs [78], species introductions [21], an expansion in aquatic weeds [23], and hydrologic and operational changes [29,79]. As noted by Feyrer et al. [75], these anthropogenic disturbances can be powerful enough to overwhelm natural processes driving estuarine variability. Hence, ecosystem shifts are often extremely difficult or impractical to reverse [1]. With the added prospect of climate change [80,81,82] and the vulnerability of the region to large scale disturbances such as earthquake and flood [83,84], it is likely that there may be additional ecosystem shifts in the foreseeable future. Given that economic uses of the Delta are closely tied with its ecological health [15], the effects of present and future ecosystem shifts are expected to radiate well beyond fish communities.

## Conclusions

The Delta has been heavily altered since the 1800s and will continue to change rapidly as Californians attempt to simultaneously manage the Delta for both water supplies and ecosystem health. We observed apparent trends of increase in the annual catch of most fish species in the beach seine survey since the late 1990s. We also found that interannual variation in the littoral fish community composition largely followed a pattern of simple progression through time over the study period, though year-to-year variability in freshwater inflow seem to play a substantial role as well. Notably, biomass per volume has also doubled over the study period, largely driven by a handful of species from the Mississippi drainage that are now widespread in the Delta [25,33]. With sea level rise and land subsidence continuing to be an issue in many Delta islands, the Delta is likely to change from the current system of tidal freshwater channels to a more diverse area consisting of flooded islands and shallow open water [37]. Ongoing and planned tidal wetland restoration efforts around the Delta would also further expand shallow water habitat [85]. As such habitat becomes more widespread, an even larger increase in littoral fish abundance seems likely. However, it remains uncertain whether these new tidal habitats would favor more desirable native fishes or the already numerous non-native fish species.

## Supporting Information

S1 Fig Map of study area with beach seine survey locations and their respective site names (for use in conjunction with S1 Table). (PDF)

S2 Fig NMDS plot of fourth-root transformed annual fish catch per effort with all species correlation vectors plotted (top) and March-August daily average freshwater inflow from DAYFLOW plotted by year (bottom). (PDF)

S3 Fig Similarity profile dendrogram results based on fourth-root transformed annual species catch per effort. Significant clusters at *p* < 0.05 are denoted by each unique colored lines.
(PDF)

S4 Fig Annual catch per effort for the 23 species used in catch trend and change-point analyses (see Fig 2). (PDF)

S1 Table Number of months between the March-August in a year that lacks a single sampling event by station and the typical substrate found at each station. Pavement substrate indicates that the site is a boating ramp. For location of each station, see S1 Fig.
(PDF)

S2 Table List of species used in each analysis and total catch numbers in the study’s dataset (March to August for 26 sites within the Delta between 1995 and 2015). (PDF)

S3 Table List of *p*-values for the Mann-Kendall tests conducted on annual catch per effort numbers (as seen in Fig 2) ordered from lowest to highest with step-up false discovery rate adjusted α. (PDF)

S4 Table List of *p*-values for the Pettitt’s tests conducted on annual catch per effort numbers (as seen in Fig 2) ordered from lowest to highest with step-up false discovery rate adjusted α. *Same *p*-values for Fathead Minnow and Mississippi Silversides were observed, thus we used the higher threshold (α) to test for significance.
(PDF)

S5 Table Mean species abundance (fourth-root transformed catch per effort) for each significant clusters assigned by way of similarity profile (SIMPROF) analysis (see S1 Fig). Only top seven most abundant species in each cluster are shown.
(PDF)

S6 Table Similarity percentage analysis results showing seven species with highest average contributions to overall dissimilarity between SIMPROF groups listed in S4 Table. (PDF)
