# Supplementary material for: Evidence of a Shift in the Littoral Fish Community of the Sacramento-San Joaquin Delta
Source: PLoS One. 2017 Jan 24;12(1):e0170683. doi: 10.1371/journal.pone.0170683 (PMC5261730; doi:10.1371/journal.pone.0170683)

S1 Fig. Map of study area with beach seine survey locations and their respective site names (for use in conjunction with S1 Table)

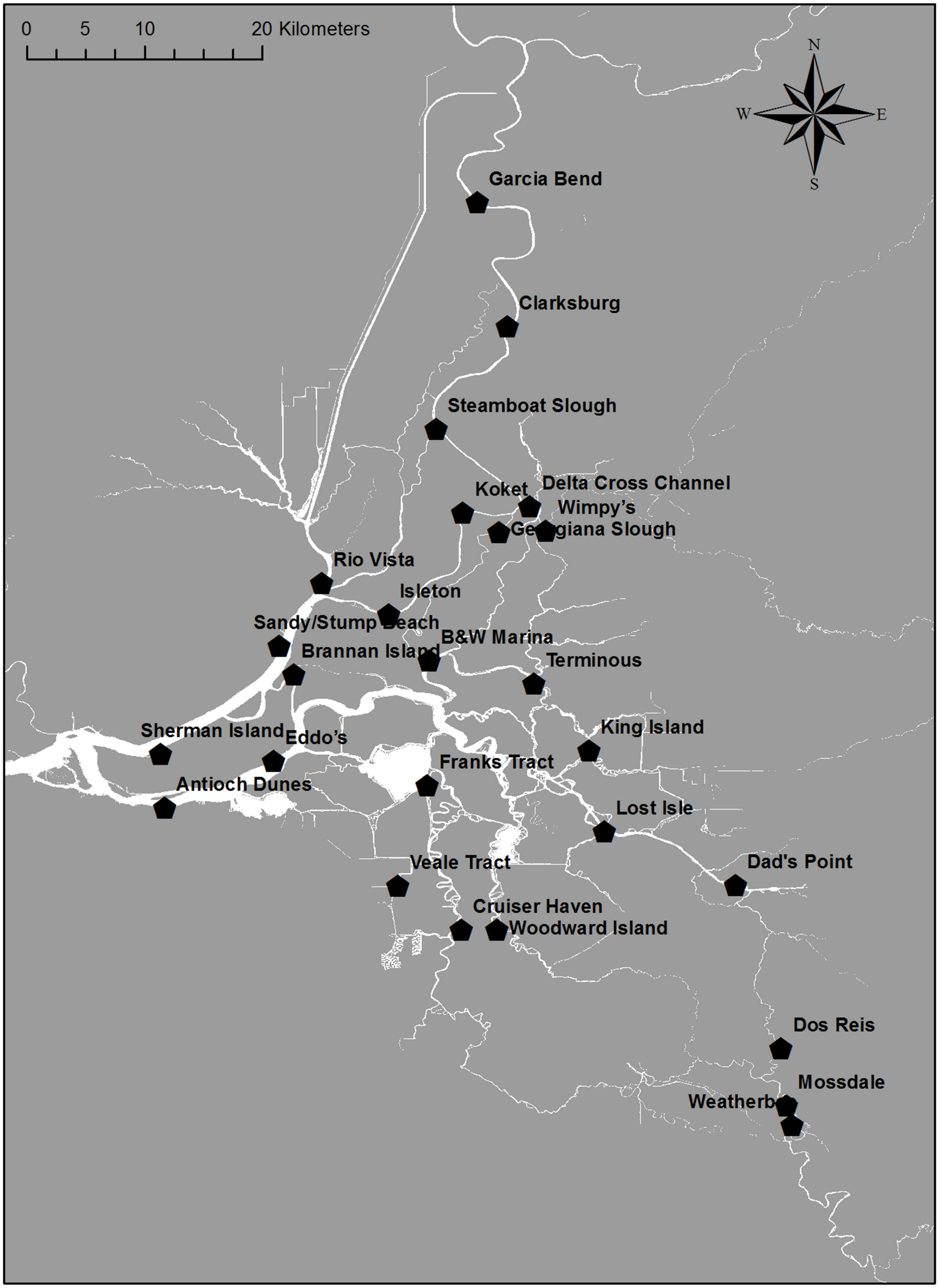

Supplement: S1 Fig — (PDF) [file pone.0170683.s001.pdf]
