# Supplementary material for: Evidence of a Shift in the Littoral Fish Community of the Sacramento-San Joaquin Delta
Source: PLoS One. 2017 Jan 24;12(1):e0170683. doi: 10.1371/journal.pone.0170683 (PMC5261730; doi:10.1371/journal.pone.0170683)

S2 Fig. NMDS plot of fourth-root transformed annual fish catch per effort with all species correlation vectors plotted (top) and March-August daily average freshwater inflow from DAYFLOW plotted by year (bottom).

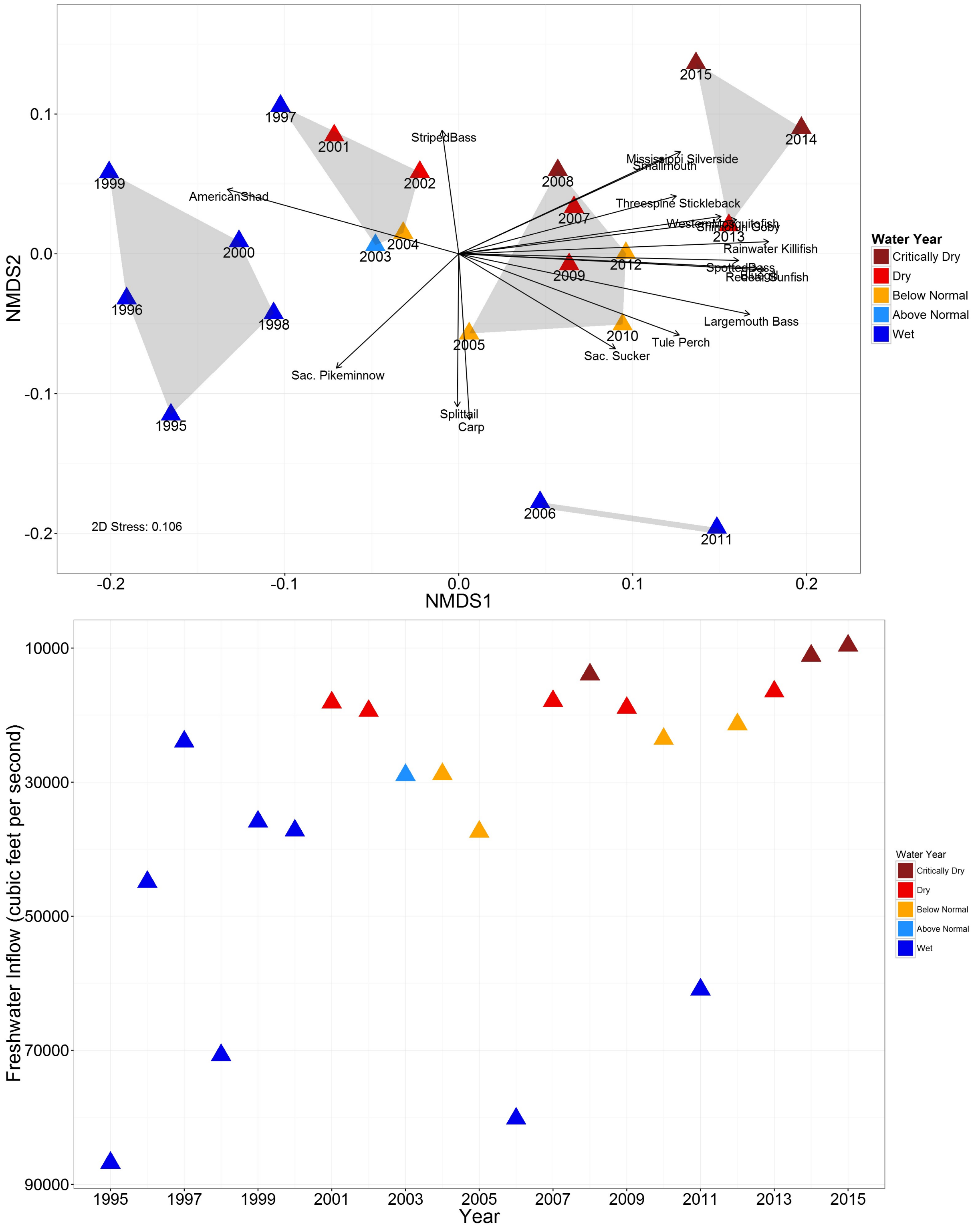

Supplement: S2 Fig — (PDF) [file pone.0170683.s002.pdf]
