# Supplementary material for: Evidence of a Shift in the Littoral Fish Community of the Sacramento-San Joaquin Delta
Source: PLoS One. 2017 Jan 24;12(1):e0170683. doi: 10.1371/journal.pone.0170683 (PMC5261730; doi:10.1371/journal.pone.0170683)

S3 Fig. Similarity profile dendrogram results based on fourth-root transformed annual species catch per effort. Significant clusters at $p < 0.05$ are denoted by each unique colored lines.

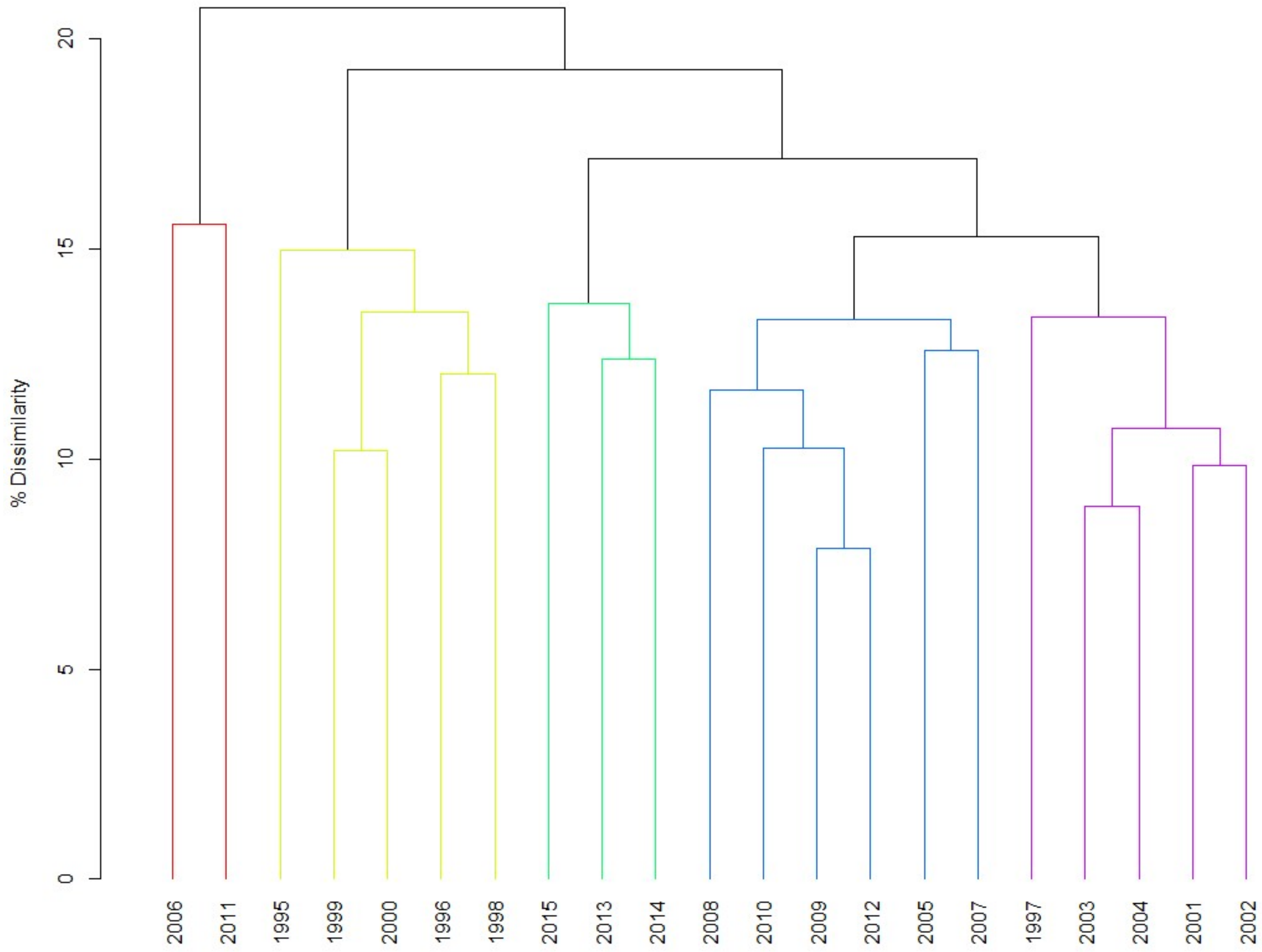

Supplement: S3 Fig — Significant clusters at p < 0.05 are denoted by each unique colored lines. (PDF) [file pone.0170683.s003.pdf]
