# Supplementary material for: Evidence of a Shift in the Littoral Fish Community of the Sacramento-San Joaquin Delta
Source: PLoS One. 2017 Jan 24;12(1):e0170683. doi: 10.1371/journal.pone.0170683 (PMC5261730; doi:10.1371/journal.pone.0170683)
Supplement: S4 Fig — (PDF) [file pone.0170683.s004.pdf]

S4 Fig. Annual catch per effort for the 23 species used in catch trend and change-point analyses (see Fig 2).

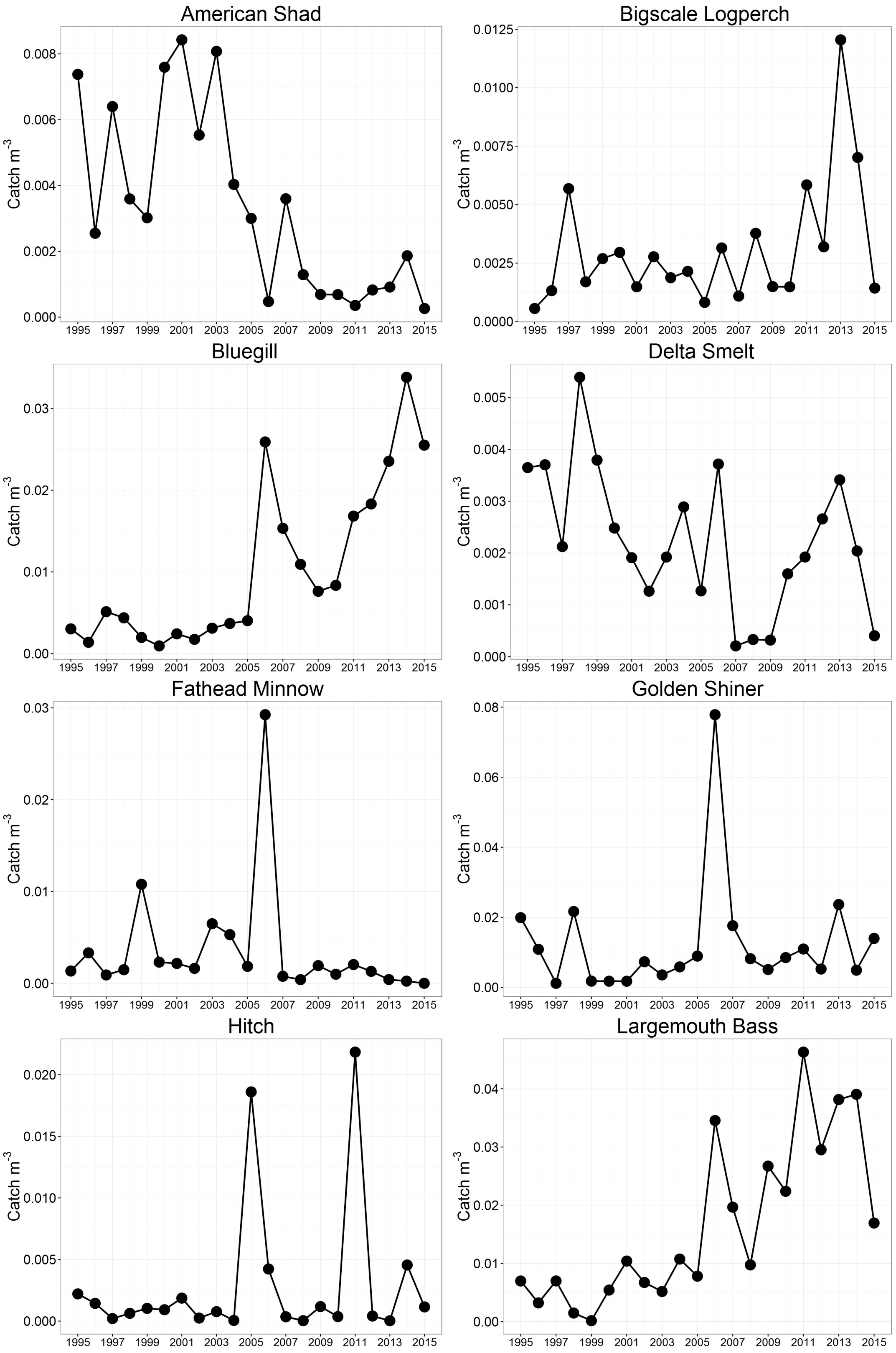

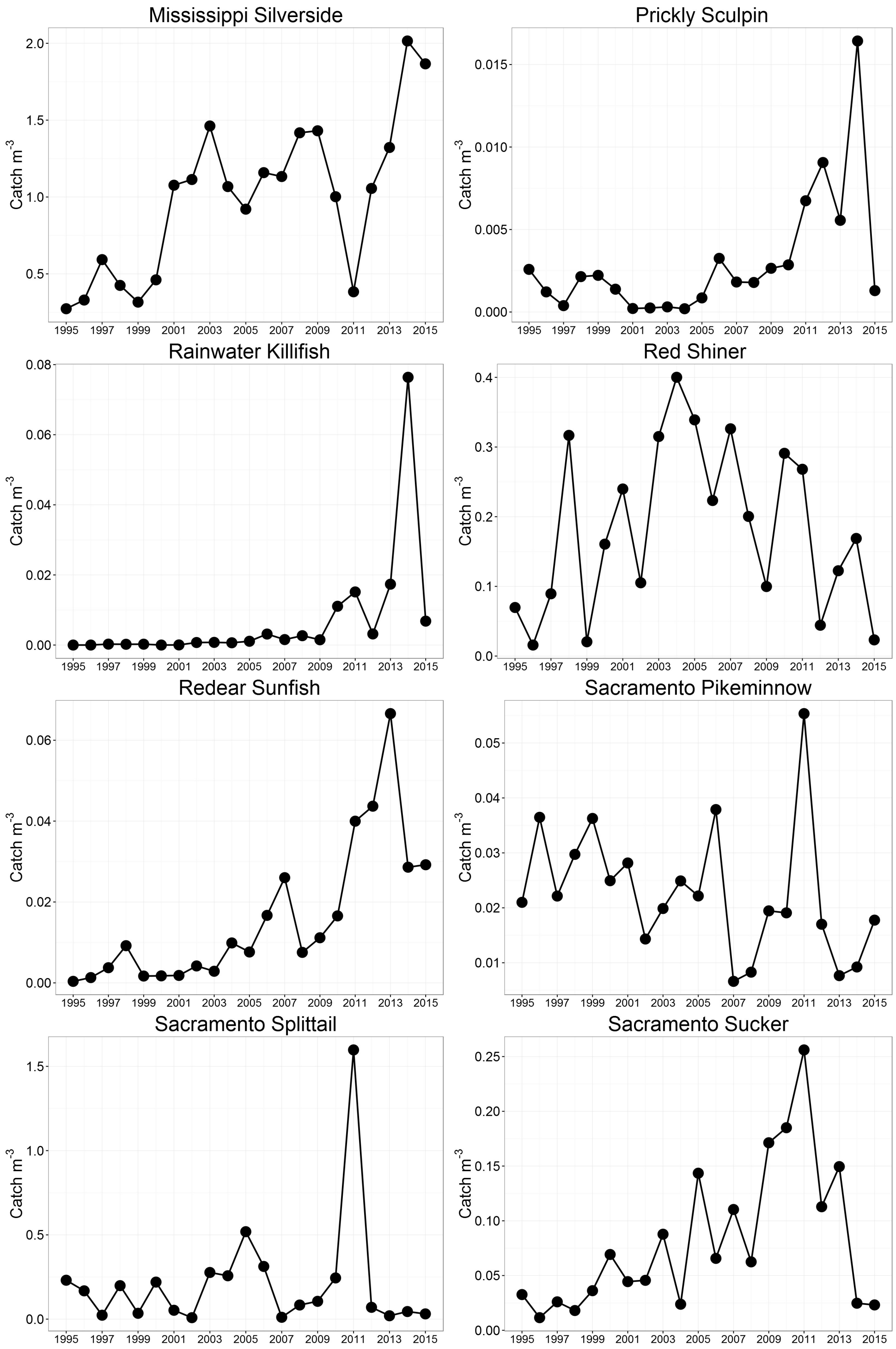
Mississippi Silverside
Prickly Sculpin
Rainwater Killifish
Red Shiner
Redear Sunfish
Sacramento Pikeminnow
Sacramento Splittail
Sacramento Sucker
Catch m-3
1995 1997 1999 2001 2003 2005 2007 2009 2011 2013 2015
0.5 1.0 1.5 2.0
0.000 0.005 0.010 0.015
0.00 0.02 0.04 0.06 0.08
0.0 0.1 0.2 0.3 0.4
0.00 0.02 0.04 0.06
0.01 0.02 0.03 0.04 0.05
0.0 0.5 1.0 1.5
0.00 0.05 0.10 0.15 0.20 0.25

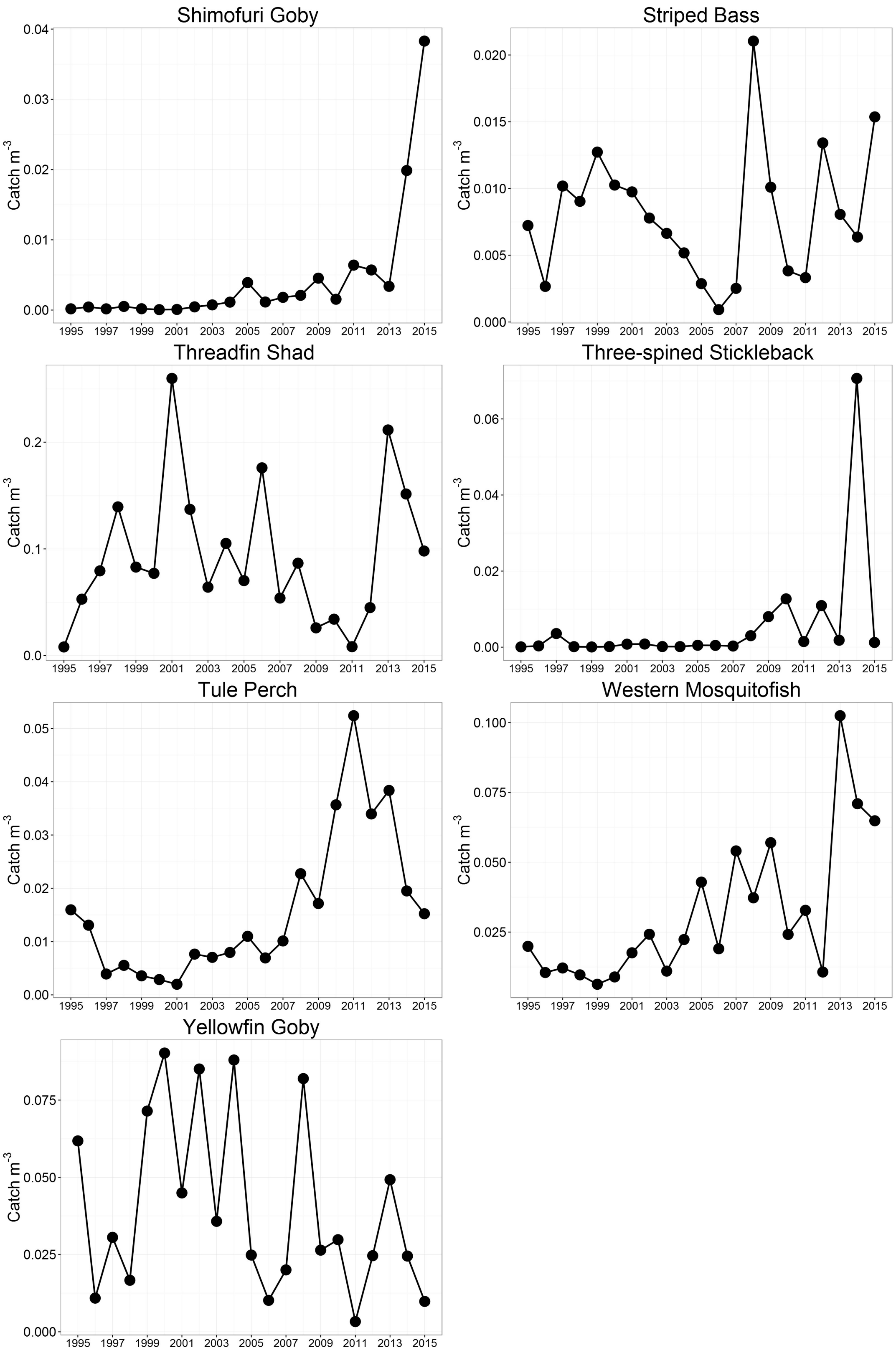
Shimofuri Goby
Catch m-3
0.04
0.03
0.02
0.01
0.00
1995 1997 1999 2001 2003 2005 2007 2009 2011 2013 2015
Striped Bass
Catch m-3
0.020
0.015
0.010
0.005
0.000
1995 1997 1999 2001 2003 2005 2007 2009 2011 2013 2015
Threadfin Shad
Catch m-3
0.2
0.1
0.0
1995 1997 1999 2001 2003 2005 2007 2009 2011 2013 2015
Three-spined Stickleback
Catch m-3
0.06
0.04
0.02
0.00
1995 1997 1999 2001 2003 2005 2007 2009 2011 2013 2015
Tule Perch
Catch m-3
0.05
0.04
0.03
0.02
0.01
0.00
1995 1997 1999 2001 2003 2005 2007 2009 2011 2013 2015
Western Mosquitofish
Catch m-3
0.100
0.075
0.050
0.025
1995 1997 1999 2001 2003 2005 2007 2009 2011 2013 2015
Yellowfin Goby
Catch m-3
0.075
0.050
0.025
0.000
1995 1997 1999 2001 2003 2005 2007 2009 2011 2013 2015
