# Supplementary material for: Evidence of a Shift in the Littoral Fish Community of the Sacramento-San Joaquin Delta
Source: PLoS One. 2017 Jan 24;12(1):e0170683. doi: 10.1371/journal.pone.0170683 (PMC5261730; doi:10.1371/journal.pone.0170683)
Supplement: S1 Table — Pavement substrate indicates that the site is a boating ramp. For location of each station, see S1 Fig. (PDF) [file pone.0170683.s005.pdf]

S1 Table. Number of months between the March-August in a year that lacks a single sampling event by station and the typical substrate found at each station. Pavement substrate indicates that the site is a boating ramp. For mapped location of each station, see S1 Fig.

Location	Substrate	Year																				
		1995	1996	1997	1998	1999	2000	2001	2002	2003	2004	2005	2006	2007	2008	2009	2010	2011	2012	2013	2014	2015
**Antioch Dunes**	Sand	-	-	-	-	-	-	-	-	-	-	-	-	-	-	-	-	-	-	-	-	-
**B&W Marina**	Pavement	-	-	-	-	-	-	-	-	-	-	-	-	-	-	-	-	-	-	-	-	-
**Brannan Island**	Sand	-	-	-	-	-	-	-	-	-	-	-	-	-	-	-	-	-	-	1	2	-
**Clarksburg**	Pavement	-	-	-	-	-	-	-	1	-	-	-	-	1	-	-	-	-	-	-	-	-
**Cruiser Haven**	Mud	1	-	-	-	-	1	-	-	-	-	-	1	-	-	-	-	-	1	-	-	-
**Dad's Point**	Sand	4	2	2	2	1	1	-	-	1	-	-	-	-	-	-	-	-	-	-	-	-
**Delta Cross Channel**	Mud	-	-	-	2	2	-	-	-	-	1	1	2	2	3	2	3	5	1	-	1	-
**Dos Reis**	Pavement	2	2	2	2	1	1	-	-	-	-	-	-	-	1	-	-	-	-	-	-	-
**Eddo's**	Pavement	-	-	-	-	-	-	-	-	-	-	-	-	-	-	-	-	-	-	-	-	-
**Franks Tract**	Sand	2	-	-	2	-	-	-	-	-	-	-	1	-	-	-	-	-	-	-	-	-
**Garcia Bend**	Pavement	-	-	1	-	-	-	-	-	-	-	-	-	1	-	-	-	-	-	-	-	-
**Georgiana Slough**	Sand/Mud	-	-	-	1	-	-	-	-	-	2	-	2	1	-	-	-	-	-	-	-	-
**Isleton**	Sand/Pavement	1	-	-	-	-	-	-	-	1	-	-	-	1	-	-	-	-	-	-	-	-
**King Island**	Pavement	-	-	-	-	-	-	-	-	-	-	-	-	-	-	-	-	-	-	-	-	-
**Koket**	Mud	-	-	-	-	-	-	-	-	-	-	-	-	1	-	-	-	-	-	-	-	-
**Lost Isle**	Sand	-	-	-	-	-	-	-	-	-	-	-	-	-	-	-	-	-	-	-	-	-
**Mossdale**	Pavement	2	2	3	2	1	1	-	-	-	-	-	-	-	-	-	-	-	-	-	-	-
**Rio Vista**	Mud	-	-	1	-	-	-	-	-	-	-	-	-	-	1	-	-	-	-	-	-	-
**Sandy/Stump Beach**	Sand	-	1	2	-	-	-	-	-	-	-	-	-	1	-	-	-	-	-	-	-	-
**Sherman Island**	Sand/Mud	-	-	-	-	-	-	-	-	-	-	-	-	-	-	-	-	-	-	-	-	-
**Steamboat Slough**	Sand/Pavement	-	-	-	-	-	-	-	-	-	-	-	1	1	-	-	-	-	-	1	-	-
**Terminous**	Mud/Sand	-	-	-	-	-	-	-	-	-	-	-	-	-	-	-	-	-	-	-	-	-
**Veale Tract**	Sand	1	-	-	-	-	2	-	1	-	-	-	1	-	-	-	2	2	-	-	2	-
**Weatherbee**	Sand	4	2	2	2	2	2	2	2	1	2	-	-	-	2	-	-	2	-	-	-	-
**Wimpy's**	Pavement	-	-	-	-	-	-	-	-	-	-	-	-	1	-	-	-	-	-	-	-	-
**Woodward Island**	Sand	1	1	1	1	-	1	-	-	-	-	-	-	-	-	-	-	-	-	-	-	-
