# Supplementary material for: Evidence of a Shift in the Littoral Fish Community of the Sacramento-San Joaquin Delta
Source: PLoS One. 2017 Jan 24;12(1):e0170683. doi: 10.1371/journal.pone.0170683 (PMC5261730; doi:10.1371/journal.pone.0170683)
Supplement: S2 Table — (PDF) [file pone.0170683.s006.pdf]

**S2 Table. List of species used in each analysis and total catch numbers in the study's dataset (March to August for 26 sites within the Delta between 1995 and 2015).**

| Q1: Species Catch Pattern | Q2: Fish Community Composition | Q3: Biomass | Latin Name | Family | Total Catch |
|---|---|---|---|---|---|
| American Shad | American Shad | American Shad | *Alosa supidissima* | Clupeidae | 3,274 |
| Bigscale Logperch | Bigscale Logperch | Bigscale Logperch | *Percina macrolepida* | Percidae | 1,263 |
| | | Black Bullhead | *Ameiurus melas* | Ictaluridae | 34 |
| | | Unknown Black Bass | *Micropterus spp.* | Centrarchidae | 255 |
| | Black Crappie | Black Crappie | *Pomoxis nigromaculatus* | Centrarchidae | 257 |
| Bluegill | Bluegill | Bluegill | *Lepomis macrochirus* | Centrarchidae | 7,274 |
| | | Brown Bullhead | *Ameiurus nebulosus* | Ictaluridae | 25 |
| | | California Roach | *Hesperoleucus symmetricus* | Cyprinidae | 32 |
| | | Chameleon Goby | *Tridentiger trigonocephalus* | Gobiidae | 33 |
| | | Channel Catfish | *Ictalurus punctatus* | Ictaluridae | 10 |
| | Common Carp | Common Carp | *Cyprinus carpio* | Cyprinidae | 1,845 |
| Delta Smelt | Delta Smelt | Delta Smelt | *Hypomesus transpacificus* | Osmeridae | 1,295 |
| Fathead Minnow | Fathead Minnow | Fathead Minnow | *Pimephales promelas* | Cyprinidae | 2,259 |
| | | Goldfish | *Carassius auratus* | Cyprinidae | 42 |
| Golden Shiner | Golden Shiner | Golden Shiner | *Notemigonus crysoleucas* | Cyprinidae | 6,730 |
| | | Green Sunfish | *Lepomis cyanellus* | Centrarchidae | 64 |
| | Hardhead | Hardhead | *Mylopharodon conocephalus* | Cyprinidae | 131 |
| Hitch | Hitch | Hitch | *Lavinia exilicauda* | Cyprinidae | 1,445 |
| Mississippi Silverside | Mississippi Silverside | Mississippi Silverside | *Menidia audens* | Atherinopsidae | 1,118,510 |
| Largemouth Bass | Largemouth Bass | Largemouth Bass | *Micropterus salmoides* | Centrarchidae | 8,345 |
| | | Longfin Smelt | *Spirinchus thaleichthys* | Osmeridae | 29 |
| | | Pacific Herring | *Clupea pallasii* | Clupeidae | 18 |
| | Pacific Staghorn Sculpin | Pacific Staghorn Sculpin | *Leptocottus armatus* | Cottidae | 908 |
| Prickly Sculpin | Prickly Sculpin | Prickly Sculpin | *Cottus asper* | Cottidae | 999 |
| Rainwater Killifish | Rainwater Killifish | Rainwater Killifish | *Lucania parva* | Fundulidae | 2,778 |
| Red Shiner | Red Shiner | Red Shiner | *Cyprinella lutrensis* | Cyprinidae | 120,282 |
| Redear Sunfish | Redear Sunfish | Redear Sunfish | *Lepomis microlophus* | Centrarchidae | 9,934 |
| | | Redeye Bass | *Micropterus coosae* | Centrarchidae | 20 |
| | Sacramento Blackfish | Sacramento Blackfish | *Orthodon microlepidotus* | Cyprinidae | 127 |
| Sacramento Pikeminnow | Sacramento Pikeminnow | Sacramento Pikeminnow | *Ptychocheilus grandis* | Cyprinidae | 11,080 |
| Sacramento Sucker | Sacramento Sucker | Sacramento Sucker | *Catostomus occidentalis* | Catostomidae | 35,149 |

| | | | | | |
|---|---|---|---|---|---|
| Shimofuri Goby | Shimofuri Goby | Shimofuri Goby | *Tridentiger bifasciatus* | Gobiidae | 2,796 |
| | | Shokihaze Goby | *Tridentiger barbatus* | Gobiidae | 3 |
| | Smallmouth Bass | Smallmouth Bass | *Micropterus dolomieu* | Centrarchidae | 325 |
| Splittail | Splittail | Splittail | *Pogonichthys macrolepidotus* | Cyprinidae | 86,258 |
| | Spotted Bass | Spotted Bass | *Micropterus punctulatus* | Centrarchidae | 1,660 |
| | | Starry Flounder | *Platichthys stellatus* | Pleuronectidae | 58 |
| Striped Bass | Striped Bass | Striped Bass | *Morone saxatilis* | Moronidae | 4,212 |
| Threadfin Shad | Threadfin Shad | Threadfin Shad | *Dorosoma petenense* | Clupeidae | 148,819 |
| Three-spined Stickleback | Three-spined Stickleback | Three-spined Stickleback | *Gasterosteus aculeatus* | Gasterosteidae | 1,120 |
| Tule Perch | Tule Perch | Tule Perch | *Hysterocarpus traskii* | Embiotocidae | 6,408 |
| | Wakasagi | Wakasagi | *Hypomesus nipponensis* | Osmeridae | 1,052 |
| | | Warmouth | *Lepomis gulosus* | Centrarchidae | 24 |
| Western Mosquitofish | Western Mosquitofish | Western Mosquitofish | *Gambusia affinis* | Poeciliidae | 13,569 |
| | | White Catfish | *Ameiurus catus* | Ictaluridae | 41 |
| | | White Crappie | *Pomoxis annularis* | Centrarchidae | 77 |
| Yellowfin Goby | Yellowfin Goby | Yellowfin Goby | *Acanthogobius flavimanus* | Gobiidae | 13,990 |
