# Supplementary material for: Evidence of a Shift in the Littoral Fish Community of the Sacramento-San Joaquin Delta
Source: PLoS One. 2017 Jan 24;12(1):e0170683. doi: 10.1371/journal.pone.0170683 (PMC5261730; doi:10.1371/journal.pone.0170683)
Supplement: S3 Table — (PDF) [file pone.0170683.s007.pdf]

S3 Table. List of *p*-values for the Mann-Kendall tests conducted on annual catch per effort numbers (as seen in Fig 2) ordered from lowest to highest with step-up false discovery rate adjusted α.

Species	*p* for Mann-Kendall test	Adjusted threshold (α)	Significant?
Rainwater Killifish	7.15×10^{-7}	0.00217	Yes
Redear Sunfish	2.86×10^{-6}	0.00435	Yes
Shimofuri Goby	2.86×10^{-6}	0.00652	Yes
Bluegill	5.91×10^{-5}	0.00870	Yes
Largemouth Bass	9.80×10^{-5}	0.01087	Yes
Western Mosquitofish	0.00100	0.01304	Yes
Mississippi Silverside	0.00100	0.01522	Yes
American Shad	0.00152	0.01739	Yes
Three-spined Stickleback	0.00152	0.01957	Yes
Tule Perch	0.00279	0.02174	Yes
Sacramento Sucker	0.01220	0.02391	Yes
Prickly Sculpin	0.02006	0.02609	Yes
Sacramento Pikeminnow	0.02750	0.02826	Yes
Fathead Minnow	0.03203	0.03043	No
Delta Smelt	0.08521	0.03261	No
Bigscale Logperch	0.10950	0.03478	No
Yellowfin Goby	0.13897	0.03696	No
Golden Shiner	0.31901	0.03913	No
Red Shiner	0.78580	0.04130	No
Sacramento Splittail	0.78580	0.04348	No
Hitch	0.87999	0.04565	No
Threadfin Shad	0.92782	0.04783	No
Striped Bass	0.97591	0.05000	No
