# Supplementary material for: Evidence of a Shift in the Littoral Fish Community of the Sacramento-San Joaquin Delta
Source: PLoS One. 2017 Jan 24;12(1):e0170683. doi: 10.1371/journal.pone.0170683 (PMC5261730; doi:10.1371/journal.pone.0170683)
Supplement: S4 Table — *Same p-values for Fathead Minnow and Mississippi Silversides were observed, thus we used the higher threshold (α) to test for significance. (PDF) [file pone.0170683.s008.pdf]

S4 Table. **List of *p*-values for the Pettitt's tests conducted on annual catch per effort numbers (as seen in Fig 2) ordered from lowest to highest with step-up false discovery rate adjusted α.** *Same *p*-values for Fathead Minnow and Mississippi Silversides were observed, thus we used the higher threshold (α) to test for significance.

Species	*p* for Pettitt's test	Adjusted threshold (α)	Significant?
Bluegill	0.00113	0.00217	Yes
Rainwater Killifish	0.00113	0.00435	Yes
Shimofuri Goby	0.00113	0.00652	Yes
Largemouth Bass	0.00192	0.00870	Yes
Redear Sunfish	0.00250	0.01087	Yes
American Shad	0.00321	0.01304	Yes
Tule Perch	0.00321	0.01522	Yes
Three-spined Stickleback	0.00670	0.01739	Yes
Prickly Sculpin	0.01335	0.01957	Yes
Western Mosquitofish	0.01335	0.02174	Yes
Fathead Minnow	0.02546	0.02391	Yes*
Mississippi Silverside	0.02546	0.02609	Yes*
Sacramento Pikeminnow	0.05620	0.02826	No
Sacramento Sucker	0.08104	0.03043	No
Delta Smelt	0.09660	0.03261	No
Yellowfin Goby	0.18561	0.03478	No
Bigscale Logperch	0.37566	0.03696	No
Golden Shiner	0.42617	0.03913	No
Red Shiner	0.48108	0.04130	No
Sacramento Splittail	0.74354	0.04348	No
Threadfin Shad	0.81884	0.04565	No
Striped Bass	0.89733	0.04783	No
Hitch	0.99999	0.05000	No
