# Supplementary material for: Evidence of a Shift in the Littoral Fish Community of the Sacramento-San Joaquin Delta
Source: PLoS One. 2017 Jan 24;12(1):e0170683. doi: 10.1371/journal.pone.0170683 (PMC5261730; doi:10.1371/journal.pone.0170683)
Supplement: S5 Table — Only top seven most abundant species in each cluster are shown. (PDF) [file pone.0170683.s009.pdf]

S5 Table. Mean species abundance (fourth-root transformed catch per effort) for each significant clusters assigned by way of similarity profile (SIMPROF) analysis (see S1 Fig). Only top seven most abundant species in each cluster are shown.

SIMPROF Cluster	Years Included	Species	Mean species abundance within cluster (fourth-root transformed catch per m^3)
A	1995, 1996, 1998, 1999, 2000	Mississippi Silverside	0.772
		Sacramento Splittail	0.623
		Red Shiner	0.526
		Threadfin Shad	0.491
		Yellowfin Goby	0.449
		Sacramento Sucker	0.414
		Sacramento Pikeminnow	0.413
B	1997, 2001, 2002, 2003, 2004	Mississippi Silverside	1.008
		Red Shiner	0.672
		Threadfin Shad	0.585
		Sacramento Splittail	0.523
		Yellowfin Goby	0.480
		Sacramento Sucker	0.452
		Sacramento Pikeminnow	0.383
C	2005, 2007, 2008, 2009, 2010, 2012	Mississippi Silverside	1.035
		Red Shiner	0.657
		Sacramento Sucker	0.595
		Sacramento Splittail	0.583
		Threadfin Shad	0.472
		Western Mosquitofish	0.430
		Yellowfin Goby	0.421
D	2006, 2011	Sacramento Splittail	0.936
		Mississippi Silverside	0.912
		Red Shiner	0.703
		Sacramento Sucker	0.609
		Common Carp	0.483
		Threadfin Shad	0.475
		Sacramento Pikeminnow	0.463
E	2013, 2014, 2015	Mississippi Silverside	1.144
		Threadfin Shad	0.621
		Red Shiner	0.541
		Western Mosquitofish	0.529
		Sacramento Sucker	0.470
		Redear Sunfish	0.444
		Sacramento Splittail	0.419
