# Supplementary material for: Evidence of a Shift in the Littoral Fish Community of the Sacramento-San Joaquin Delta
Source: PLoS One. 2017 Jan 24;12(1):e0170683. doi: 10.1371/journal.pone.0170683 (PMC5261730; doi:10.1371/journal.pone.0170683)
Supplement: S6 Table — (PDF) [file pone.0170683.s010.pdf]

S6 Table. Similarity percentage analysis results showing seven species with highest average contributions to overall dissimilarity between SIMPROF groups listed in S4 Table.

SIMPROF group comparison	Species	Mean abundance for 1st group	Mean abundance for 2nd group	Ordered cumulative contribution to dissimilarity (%)
A-B	Mississippi Silverside	0.772	1.008	9.2
	Red Shiner	0.526	0.672	16.8
	Sacramento Splittail	0.624	0.523	24.0
	Common Carp	0.153	0.057	28.6
	Threadfin Shad	0.490	0.585	33.2
	Spotted Bass	0	0.110	37.4
	Sacramento Blackfish	0.122	0.013	41.7
A-C	Mississippi Silverside	0.773	1.035	7.7
	Red Shiner	0.526	0.657	13.4
	Spotted Bass	0	0.192	19.0
	Sacramento Sucker	0.414	0.595	24.3
	Rainwater Killifish	0.049	0.228	29.6
	Sacramento Splittail	0.624	0.583	34.4
	Redear Sunfish	0.209	0.356	38.8
A-D	Common Carp	0.153	0.483	7.8
	Sacramento Splittail	0.624	0.936	15.1
	Rainwater Killifish	0.049	0.294	20.9
	Largemouth Bass	0.221	0.448	26.2
	Red Shiner	0.526	0.704	30.8
	Sacramento Sucker	0.414	0.609	35.5
	Redear Sunfish	0.209	0.403	40.0
A-E	Mississippi Silverside	0.773	1.144	8.4
	Rainwater Killifish	0.049	0.392	16.0
	Spotted Bass	0	0.242	21.4
	Redear Sunfish	0.209	0.444	26.7

	Shimofuri Goby	0.123	0.353	32.0
	Western Mosquitofish	0.320	0.529	36.6
	Sacramento Splittail	0.624	0.419	41.3
B-C	Sacramento Splittail	0.523	0.583	7.4
	Sacramento Sucker	0.452	0.595	12.7
	Pacific Staghorn Sculpin	0.145	0.259	17.8
	Wakasagi	0.063	0.181	22.7
	Red Shiner	0.672	0.657	27.1
	Threadfin Shad	0.585	0.472	31.3
	Redear Sunfish	0.251	0.356	35.3
B-D	Common Carp	0.057	0.483	10.0
	Sacramento Splittail	0.523	0.936	19.7
	Yellowfin Goby	0.480	0.279	24.4
	Golden Shiner	0.240	0.426	28.8
	Threadfin Shad	0.585	0.475	33.1
	Hitch	0.141	0.320	37.3
	Sacramento Sucker	0.452	0.609	41.2
B-E	Rainwater Killifish	0.139	0.392	7.1
	Shimofuri Goby	0.141	0.353	13.3
	Redear Sunfish	0.251	0.444	18.8
	Bluegill	0.235	0.407	23.7
	Western Mosquitofish	0.360	0.529	28.5
	Red Shiner	0.672	0.541	33.2
	Sacramento Splittail	0.523	0.419	37.8
C-D	Common Carp	0.102	0.483	10.6
	Sacramento Splittail	0.583	0.936	20.9
	Pacific Staghorn Sculpin	0.259	0.105	25.8
	Threadfin Shad	0.472	0.475	30.6
	Hitch	0.174	0.320	35.2

	Hardhead	0.053	0.211	39.6
	Mississippi Silverside	1.035	0.912	43.6
C-E	Sacramento Splittail	0.583	0.419	6.5
	Red Shiner	0.657	0.541	12.1
	Rainwater Killifish	0.228	0.392	17.6
	Sacramento Sucker	0.595	0.470	22.6
	Threadfin Shad	0.472	0.621	27.5
	Three-spined Stickleback	0.245	0.303	32.3
	Wakasagi	0.181	0.045	37.1
D-E	Sacramento Splittail	0.936	0.419	12.0
	Common Carp	0.483	0.039	22.3
	Mississippi Silverside	0.912	1.144	27.7
	Fathead Minnow	0.313	0.089	33.0
	Threadfin Shad	0.475	0.621	37.2
	Sacramento Sucker	0.609	0.470	41.3
	Red Shiner	0.704	0.541	45.2
